# Demographic Characteristics of Unvaccinated Asymptomatic and Symptomatic SARS-CoV-2 Cases in Barwon South West, Victoria, Australia

**DOI:** 10.3390/pathogens12121420

**Published:** 2023-12-04

**Authors:** Shinae C. Tobin-Salzman, Darcie Cooper, Bridgette J. McNamara, Eugene Athan, Catherine M. Bennett

**Affiliations:** 1 Institute for Health Transformation, Deakin University, Geelong, VIC 3220, Australia; shinaects@gmail.com; 2 Centre for Innovation in Infectious Disease and Immunology Research (CIIDIR), Geelong, VIC 3220, Australia; darcie.cooper@deakin.edu.au (D.C.); bridgette.mcnamara@barwonhealth.org.au (B.J.M.); e.athan@deakin.edu.au (E.A.); 3 IMPACT, The Institute for Mental and Physical Health and Clinical Translation, Geelong, VIC 3220, Australia; 4 School of Medicine, Deakin University, Geelong, VIC 3220, Australia; 5 Barwon South West Public Health Unit, Geelong, VIC 3220, Australia; 6 Centre for Epidemiology and Biostatistics, University of Melbourne, Melbourne, VIC 3008, Australia; 7 Department of Infectious Diseases, Barwon Health, University of Geelong, Geelong, VIC 3220, Australia

**Keywords:** SARS-CoV-2, asymptomatic, COVID-19, symptom profile, demographic, testing, ethnicity

## Abstract

We investigated 328 SARS-CoV-2 cases in Barwon South West, Victoria, Australia, in the 2020 pre-vaccination period, comparing infections with symptoms to those that remained asymptomatic. De-identified self-reported data on case characteristics and symptom progression from three sequential questionnaires were examined. Multivariable logistic regression was used to model associations between demographic profiles and symptoms. Asymptomatic infections were more than three times as likely to be seen in ethnic minority groups than the Caucasian population after adjusting for gender and age [OR 3.2, 95% CI 1.5–6.7, *p* < 0.01] and were more common among cases of Asian background [OR 2.8, 95%CI 1.2–6.4]. Asymptomatic infections were also more common in youth and younger adults, but cases were approximately seven times more likely to be in seniors (≥65 years) compared with those 24 years of age or younger after adjusting for sex and ethnicity [OR 6.9, 95% CI 1.3–35.8]. The overrepresentation of ethnic minority groups among asymptomatic infections is suggestive of genetic haplotype variability by ethnic group, conferring greater cross-protection from other coronaviruses in the initial phase of the COVID-19 pandemic. Replication of this analysis in the post-vaccination era and reassessment of symptom expression according to ethnicity in a community with established vaccine and infection-induced immunity would determine whether this is a sustained association or one confined to the early stages of a pandemic in an immunologically naive population. These findings may, in part, reflect differences in testing patterns by ethnicity and true differences in disease expression, both of which are important to understand in order to inform transmission prevention strategies and tailored risk messaging according to ethnic background.

## 1. Introduction

Asymptomatic SARS-CoV-2 cases are a significant public health concern for managing disease outbreaks [1]. The absence of symptoms in asymptomatic cases contributed to contact-tracing and infection screening challenges amidst the coronavirus-disease-2019 (COVID-19) pandemic [2], with many of these infections remaining silent in the community unless identified through active case-finding via contact-tracing or workplace and healthcare screening. 

SARS-CoV-2 is the pathogen causing COVID-19. It can exhibit different symptom profiles in hosts [3]. For this study, we compared symptomatic infections (cases which return positive nucleic acid tests (PCR) for SARS-CoV-2 and display clinical symptoms throughout their infection period) or asymptomatic (symptom-free infections where a positive PCR result was returned, no previous symptoms had been reported, and no symptoms were reported throughout the isolation period or until such time as a negative test result was returned) [4,5].

Asymptomatic infection in 2020 to 2021 was more prevalent in children and young adults compared to the elderly, while symptomatic infection was more common in older adults and the elderly [6,7]. A systematic review subgroup analysis of eight studies revealed 318 asymptomatic cases in China; 49.6% were children under 18 years, 30.3% were adults aged 19 to 50 years, and only 16.9% were elderly aged 51 years plus [7]. Age, therefore, is an important consideration in symptom-based screening interventions or testing strategies worldwide. 

Furthermore, another study from 2020 concludes that the non-homogeneous incidence of SARS-CoV-2 infections across the State of Victoria in Australia in 2020 may be due to varying socio-economic circumstances [8]. This highlights that such factors should be taken into account for screening programs and public health policies to support pandemic mitigation strategies [8].

Interim phase III trial data for the AstraZeneca vaccine, the only trial to actively test participants to detect any infection regardless of symptoms, reported no change in asymptomatic SARS-CoV-2 infection incidence upon receipt of the vaccine, while symptomatic infection rates fell [9]. This raised the question of how the profile of asymptomatic infections and infections overall might change in a post-vaccine population. The number of asymptomatic infections in the phase III trials was small, and therefore, the degree to which asymptomatic cases persist or are prevented post-vaccination is unclear and may vary by demographics across populations. Identifying relationships between demographic factors and symptoms could, therefore, have important outbreak management implications, including informing active screening processes to reduce silent transmission in the community. 

In this study, we analyze COVID-19 outbreak data in Australia before vaccinations were introduced to see if it is possible to distinguish asymptomatic SARS-CoV-2 cases based on social and demographic characteristics [5]. A recent study postulated certain HLA genes may mediate asymptomatic COVID-19 infections, suggesting population genetic differences in propensity to contract an infection or symptom development once infected [10]. In particular, the authors postulate that HLA haplotypes may alter the T-cell function, increasing immune memory from other coronavirus infections and providing cross-immunity to SARS-CoV-2 on first exposure [10]. We were then interested to see whether this might translate to variation in the frequency of asymptomatic infections according to ethnic group. 

Predictors of symptom profiles are crucial to our understanding of risk in the context of both population-level outbreak control and infection management, helping to identify those most at risk of developing severe disease. The likelihood of being infectious in the absence of symptoms is also important in public health when managing outbreaks and minimizing the inadvertent spread of pathogens from asymptomatic carriers [11]. 

This study examines COVID-19 symptom profiles according to case demographics in order to characterize the patient cohorts most likely to be symptomatic or asymptomatic to determine if ethnicity may play a role in moderating risk in infections following first exposure to a novel coronavirus. A secondary objective was to investigate associations between the development of symptoms and the circumstances of the infection exposure, whether within the household or workplace, where known. 

## 2. Materials and Methods

This study, an observational case series, has three aims: to provide comprehensive demographic profiles of pre-vaccinated SARS-CoV-2 cases according to symptoms, to evaluate if associations exist between exposure type and symptoms, and to determine if certain demographics are associated with asymptomatic infection. The data represent SARS-CoV-2 cases detected in the second COVID-19 wave from the Geelong and greater Barwon region of Victoria, Australia.

The data source for this study was the Barwon Health (BH) and Deakin University (DU) COVID-19 Research Task Force and Cohort Study. Data were gathered from three patient follow-up care report forms of consenting cases who had been swabbed at BH testing sites and tested positive by PCR for COVID-19 during the second COVID-19 wave between June and August 2020. 

Cases entered the testing process via four pathways: tested as close contacts of known cases during contact tracing; passively presenting for testing because of symptoms; recommended to test having been present at identified public exposure sites where a case had been whilst infectious; or through workplace screening. After removing ineligible cases (*n* = 43) where symptom category could not be determined (missing test date and/or symptom details) or cases did not consent to be included, data from a total of 328 consenting cases who were symptomatic (*n* = 265) and asymptomatic (*n* = 63) were available for analysis. 

The de-identified sample was drawn from BOSSnet by BH researchers to comprise the final dataset. A password-protected file was maintained by DU, which included de-identified cases with the following demographics: sex, age, ethnicity, occupation, living situation, smoker status, comorbidity, and exposure type.

Multiple imputation and other methods to address missing data may introduce errors if an inadequate understanding or erroneous application of such techniques occurs, especially in the context of a novel disease [12,13,14,15,16]. Missing data rates were low, and excluding missing data was deemed the most suitable approach in this study. 

All statistical analyses were conducted using Stata, Version 17 [12]. Duplicated responses were removed, and several new variables were generated, including a categorical age variable to align WHO age classifications and included ‘youth’ (18–24 years), ‘young adult’ (25–44 years), ‘adult’ (45–64 years), and ‘senior’ (65+ years) [13]. To create subgroups of sufficient size for analysis, ethnicity was categorized into ‘ethnic majority’ (Caucasian) and ‘ethnic minority’ (other ethnicities) for initial investigation and then stratified into the most common ethnic groups for further analysis. 

The binary symptom profile variable was created by crosschecking symptom data at the test date with symptom reports throughout the monitoring period. This enabled us to identify infections as “symptomatic” that were pre-symptomatic at the time of the initial PCR test but for which symptoms were subsequently reported. These were then classified as symptomatic infections in the analyses. 

Logistic regression was used to model the relationship between demographic characteristics and symptoms, with the absence of symptoms set as the outcome model. Descriptive statistics were produced with associated 95% confidence intervals. Associations between all variables and symptom absence were examined using univariable logistic regression, and variables with an association reaching a level of significance of 0.05 were included in the multivariable models. Sex was also included in the final model, given the well-documented association with SARS-CoV-2 infection outcomes [6,7]. The relationship between ethnic background and asymptomatic infection was also analyzed.

## 3. Results

### 3.1. Sample Demographic Profile

Table 1 presents the demographic profile for the 328 SARS-CoV-2 cases, stratified by symptom presence; 265 symptomatic 80.8% (95%CI 76.1–84.9) and 63 asymptomatic (19.2% (95% CI 15.1–23.9). The different totals across some variables reflect missing values for variables. This particularly impacted additional variables not routinely collected from people presenting for COVID-19 testing, with household type, smoking status, living arrangements, and ethnicity all impacted to varying degrees. The mean age of cases was similar across symptom groups: 37.8 years [95% CI 37.2–49.1] for asymptomatic cases and 38.1 [95% CI 57.4–79.9] for symptomatic. However, young adults between 25 and 44 years accounted for a greater proportion of asymptomatic cases (69.8%, 95% CI 57.4–79.9) than symptomatic (43%, 95% CI 37.2–49.1), and had higher rates of asymptomatic disease than adults (27.9% (95% CI 21.0–35.5 and 8.4% (95% CI 3.7–15.9, respectively). There was no association apparent between sex and the presence of symptoms; however, asymptomatic cases were more likely to be from ethnic minority groups [65%, 95% CI 52.2–76.0] compared with symptomatic cases (33.7%, 95% CI 27.4–40.5). 

Healthcare workers were less likely to have symptomatic infections with 5.5% (0.7–18.7), with the highest rate of 40.6% (28.5–53.6) reported amongst those who did not have an occupation recorded. There were similar proportions of asymptomatic infections among cases who reported comorbidities and those who reported none. The majority of cases had never smoked, and this was true in both symptomatic (55.6%, 95% CI 45.6–65.1) and asymptomatic (90.9%, 95% CI 55.6–98.8) groups. All former smokers were symptomatic in this sample. 

Lastly, similar proportions of symptomatic and asymptomatic cases acquired their infection across all potential exposure settings. For example, workplace exposure accounted for 35.5% (95% CI 28.9–42.8) of symptomatic cases and 34% (95% CI 22.0–48.6) of asymptomatic cases.

### 3.2. Demographic Profile of SARS-CoV-2 Cases and Predicting Asymptomatic Infection

Table 2 displays the results from the crude and adjusted logistic regression models fitted for asymptomatic SARS-CoV-2 infection. Associations between demographic characteristics and asymptomatic infection in the univariable analysis included age, ethnicity, and occupation. Young adults (25–44 years) were three times more likely to be asymptomatic [OR 3.1, 95% CI 1.3–7.3, *p* < 0.01] compared to those aged 18–24 years. SARS-CoV-2 infections in people of Asian background were more likely to be asymptomatic [OR 3.0, 95% CI 1.4–657, *p* < 0.001] than the Caucasian patient group, and similar trends were seen among Middle Eastern and African patients. The groups for which ethnicity was unknown, or where only small numbers were represented from a country, also showed a significantly higher likelihood of having an asymptomatic infection than the Caucasian reference group (4,7, 95% CI 2.2–10.1).

The likelihood of having an asymptomatic infection was higher in essential service workers and residential aged-care workers compared with healthcare workers, but the difference was not statistically significant. Occupation and age group showed collinearity, as expected, with only the association between “other occupation unknown” and symptom absence remaining statistically significant after adjustment for age, so occupation was excluded from further multivariable analysis. There were no significant differences detected in the likelihood of presenting asymptomatic with respect to sex, comorbidity status, living situation, smoker status, or infection exposure location.

In the final multivariable logistic regression model, age and ethnicity remained significant covariates of asymptomatic SARS-CoV-2 infection independent of sex. However, the strength of the association varied. Ethnicity remained the most significantly associated covariate of asymptomatic infection, with the odds of being an asymptomatic case approximately three times greater for patients from an ethnic minority background [OR 3.4, 95% CI 1.7–6.6, *p* < 0.001]. Table 2 includes the model with patient background stratified for the more commonly represented ethnicity groups. Amongst ethnic groups, asymptomatic patients were more than twice as likely to be from an Asian background (OR 2.5, 95%CI: 1.1–5.9, *p* = 0.038).

Whist asymptomatic infections were twice as likely to be reported in young adults as in those under 18 (OR 2.2, 95%CI 0.8–5.8), the only association that remained significant in the final model was the 10-fold greater odds of being an older adult (above 65 years) if asymptomatic compared with those 24 or younger [OR 10.1, 95% CI 1.8–56.64, *p* < 0.01] after adjusting for ethnicity and sex. Given the small numbers, we examined the original individual records for the 12 individuals in the “seniors” group. We found all of those with asymptomatic infections were 70 years or older, while those with symptoms were between 65 and 68 years of age.

### 3.3. Ethnicity and Asymptomatic Infection

Ethnic minority was a significant covariate in the model for presenting with an asymptomatic SARS-CoV-2 infection. Overall, a significantly greater proportion of infections in people of ethnic minority backgrounds were asymptomatic (36.8%, 95% CI 52.2–76.0) compared to ethnic majority cases (13.7%, 95% CI 24.0–47.9). 

Table 3 provides a more detailed breakdown by ethnic group. Compared with Caucasian cases, all other groups had higher rates of asymptomatic infection, particularly Northeast Asians (41.2%, 95% CI 2.6–13.7). 

## 4. Discussion

This study makes an original contribution to our understanding of the distribution of symptom profiles in the pre-vaccine phase of the COVID-19 pandemic in Australia through analysis of SARS-CoV-2 cases where active patient follow-up permitted differentiation between pre-symptomatic and asymptomatic infections. This has allowed the characterization of infections according to symptom profiles and the investigation of association with certain demographic characteristics. This assessment has rarely been possible in previous studies due to the lack of symptom follow-up of infections in the community setting.

The overall proportion of asymptomatic and symptomatic cases of 19.2 percent observed in this cohort is consistent with previous population data [16]. The more detailed demographic analysis in this sample found symptom presentation differed according to ethnicity. This has been reported elsewhere in relation to greater symptom severity when symptoms occur [17]. However, in this instance, we report culturally and linguistically diverse groups may also have a higher rate of asymptomatic infections. Further, previous research has reported an excess burden of SARS-CoV-2 infection in people from varied cultural and linguistic backgrounds, for reasons that are not fully explained by underlying health issues or the environments in which they reside or receive care [18]. 

We also found that young adults were more likely to be asymptomatic compared to older adults in this sample; however, after adjustment for sex and ethnicity, the only age association that remained independently associated with the absence of symptoms was the patient being in the “senior” age group, 65 years and older. This appears to conflict with numerous studies reporting higher asymptomatic infection prevalence in young adults compared to the elderly [6,7]. However, on closer analysis, we found all asymptomatic infections in this age group were in cases aged over 70, while the symptomatic patients ranged in age from 65 to 68 years, with a mean age difference of 6.1 years (95%CI 3.7–8.5, *p* < 0.001) between those with symptoms and those without. This is consistent with reports of higher asymptomatic presentations of infections in older populations over 75 years or with comorbidity, where 65–68% of cases failed to develop symptoms [19,20].

It is also possible that older close contacts of known cases may have been more fearful of the possible consequences of infection and, therefore, more likely to be tested for infection without waiting for symptoms to develop. However, the active contact tracing that was in place during this phase of the pandemic would limit the risk of this bias. Nonetheless, these results should be interpreted with caution given the small sample of older patients and the range of factors that can influence testing and case reporting [16]. 

It is also important to note that infection rates may have varied through the study period, especially among children and adolescents, after the population focus of outbreaks moved from returned travelers to community-wide exposure. Subsequently, the proportion of asymptomatic SARS-CoV-2 infections may have also risen, leading to more silent spread in the community, given the evidence that asymptomatic cases are responsible for downstream transmission [21,22,23]. 

This study also found most asymptomatic infections (65%) in this sample were more often reported in people from culturally and linguistically diverse (CALD) backgrounds, and conversely, most symptomatic cases (63%) were Caucasian. Previous studies found increased severe SARS-CoV-2 infection risk among ethnic minorities when compared to white counterparts [4,5,6,7,8,9,10,11,12,13,14,15,16,17,18,19,20,21,22,23,24,25,26] but do not report on asymptomatic infections due to the inherent difficulties in distinguishing between pre-symptomatic and asymptomatic infections in the absence of follow-up data. While we were able to identify that a higher proportion of SARS-CoV-2 infections were asymptomatic among ethnic minority cases, we did not examine the severity of symptoms and therefore cannot confirm whether the symptoms that were experienced were comparatively more severe in those with clinical disease compared to the Caucasian cohort. 

Furthermore, people from ethnic minority backgrounds in the Barwon region were more likely to be in insecure employment and, therefore, may have been less likely to get tested even if they had symptoms or were prompted to do so by the health department as a close contact [8]. A positive result could lead to exclusion from work if unable to work from home [8]. On the other hand, if people from ethnically diverse backgrounds were more likely to be essential workers, then more asymptomatic infections could have been detected through workplace screening that was conducted regardless of symptoms, leading to higher asymptomatic infection ascertainment in this group. 

During this period, many SARS-CoV-2 cases among CALD groups in the Barwon region were also associated with large workplace outbreaks [4]. Because people from CALD backgrounds are more likely to experience broader related testing barriers compared to Caucasian cases [26], infection ascertainment is likely to vary between these subgroups depending on their occupation. For example, although interpreters and communication with community leaders were utilized, there remained significant challenges in building trust among workers of minority ethnicities in some circumstances, where complex household arrangements, insecure casual employment, and fears relating to visa status or other residency issues were prevalent [4]. As such, some workers from ethnic population groups may have remained reluctant to share symptom information or may have found understanding messaging related to testing and other outbreak mitigation strategies more challenging than the Caucasian majority [25]. Consequently, asymptomatic cases may be more likely to be reported through workplace screening or active contact testing in CALD communities, while both symptomatic and asymptomatic cases may be more likely to be underreported outside the workplace setting. Such cultural complexities suggest that SARS-CoV-2 symptom presentation and its association with ethnic minorities may be influenced by multiple social, cultural, and economic factors [26,27,28,29]. 

While the Barwon survey data provide a unique opportunity to monitor symptom development and determine the profile of asymptomatic cases without the complication of misclassification of pre-symptomatic infections as asymptomatic, the sample size does limit the precision and power in these analyses. We found statistically significant differences in univariable and multivariable modeling. Still, the confidence intervals demonstrate the range of values that remain plausible for these associations, limiting the practical significance of the results based on this cohort alone. Further, unmeasured confounders could be implicit in reporting occupation as ‘unknown’, which might differ according to ethnicity. Therefore, associations must be interpreted with caution.

Low numbers in some subpopulation groups may also render results unrepresentative of the general population and reduce external validity; however, given the completeness of case follow-up at this time with active border closures and strict measures in place, case ascertainment was high, and the cases in this cohort are likely to be representative of infections present in the community at that time. The study is also unlikely to be limited by possible symptom recall bias and the potential misclassification of mild symptomatic cases as asymptomatic, given the prospective nature of the surveys reduces this risk [30]. The heterogeneity in how cases classify their symptom presence or severity can make it difficult to accurately distinguish asymptomatic from presymptomatic and mild symptomatic infection [23]. However, as stated, following patients prospectively minimized recall error and reduced the risk of misclassifying someone as asymptomatic who did go on to develop symptoms sometime after their initial positive COVID-19 test.

The impact of selection bias is an important consideration in the interpretation of the findings. Cases in this study were derived from four separate pathways: clinical presentation, self-presentation to testing clinics if symptomatic, outbreak management, close contact screening, and workplace screening. Close contact follow-up and workplace testing are active screening processes and, alongside symptom-based testing, are likely to capture the majority of symptomatic and asymptomatic infections. 

## 5. Conclusions

Overall, we found that asymptomatic SARS-CoV-2 infection may be associated with ethnic background, with CALD groups reporting higher levels of asymptomatic infection. While further research is required to understand the complex interaction between ethnicity, social behavior, testing patterns, and symptom presentation, ethnicity-based differences in the likelihood of an infection remaining asymptomatic would align with the emerging view that certain genetic haplotypes might infer cross-protection from historic infections with more distantly related coronaviruses [10]. If so, this might partly offset the population-level impact of the documented increased risk of severe disease in people of CALD background who have COVID-19 clinical disease, with this risk potentially also related to genetic or biological factors, as well as structural societal and economic disparities that can exacerbate infection outcomes. 

## Figures and Tables

**Table 1:** SARS-CoV-2 cases demographic characteristics, stratified by symptom profile (*n* = 328).

Demographics	Count			% (95% CI)	
	S	A	Total	S	A
**Total**	265	63	328	80.8% (76.1–84.9)	19.2% (15.1–23.9)
**Age** _(years) (S = 265) (A = 63)_					
Youth ^(18–24 yrs)^	56	7	63	88.9% (78.4–95.4)	11.1% (4.6–21.6)
Young adult ^(25–44)^	114	44	158	**72.2% (64.5–79.0)**	**27.9% (21.0–35.5)**
Adult ^(45–64)^	87	8	95	**91.6% (84.1–96.3)**	**8.4% (3.7–15.9)**
Senior ^(65+)^	8	4	12	66.7% (34.9–90.0)	33.3% (9.9–65.1)
**Sex** _(S = 265) (A = 63)_					
Male	138	37	175	78.8% (72.1–84.7)	21.1% (15.3–27.9)
Female	127	26	153	83.0% (76.1–88.6)	17.0% (11.4–23.9)
**Ethnicity** _(S = 199) (A = 60)_					
Caucasian	132	21	153	**86.3% (79.8–91.3)**	**13.7% (8.7–20.2)**
Southeast Asian	18	7	25	72.0% (50.6–87.9)	28.0% (12.1–49.4)
Northeast Asian	10	7	17	58.8% (13.9–81.6)	41.2% (18.4–67.1)
Southern and Central Asian	5	2	7	71.4% (29.0–96.3)	28.6% (3.7–71.0)
African	6	3	9	66,7% (29.9–92.5)	33.3% (7,5–70.1)
Middle Eastern	4	2	6	66.7% (22.3–95.7)	33.3% (4.3–77.7)
Other	24	18	42	**57.1% (41.0–72.2)**	**42.9% (27.7–59.0)**
**Occupation** _(S = 222) (A = 58)_					
Healthcare worker	34	2	36	**94.4% (81.3–99.3)**	**5.5% (0.7–18.7)**
Essential service worker	55	14	69	79.7% (68.3–88.4)	20.3% (11.6–31.7)
Residential age care worker	15	2	17	88.2% (63.6–98.5)	11.8% (1.5–36.4)
Other working	67	9	76	**88.2% (78.7–94.4)**	**11.8% (5.6–21.3)**
Other not working	13	5	18	72.2% (46.5–90.3)	27.8% (9.7–53.5)
Other unknown	38	26	64	**59.4% (43.4–71.5)**	**40.6% (28.5–53.6)**
**Living** _(S = 254) (A = 63)_					
Family	197	36	233	**84.5% (79.3–88.9)**	**15.5% (11.1–20.7)**
Friends/housemates	46	24	70	**65.7% (53.4–76.7)**	**34.3% (23.2–46.6)**
Alone	11	3	14	78.6% (49.2–95.3)	21.4% (4.7–50.8)
**Comorbidity** _(S = 265) (A = 63)_					
Yes	33	7	40	82.5% (67.2–92.7)	17.5% (7.3–32.8)
None reported	232	56	288	80.6% (75.5–85.0)	19.4% (15.0–24.5)
**Smoker status** _(S = 99) (A = 11)_					
Never	55	10	65	84.6% (73.5–92.4)	15.3% (7.6–26.5)
Current	12	1	13	92.3% (64.8–99.8)	7.7% (0.2–36.0)
Former	32	0	32	100% (89.1–1.0 *)	0.0%
**Exposure location** (S = 183) (A = 47)					
Household	95	22	117	81.2% (72.9–87.8)	18.8% (12.2–27.1)
Workplace	65	16	81	80.2% (69.9–82.3)	19.8% (11.7–30.1)
Other	23	9	32	71.9% (53.3–86.3)	28.1% (13.7–46.7)

Note. CI: confidence interval; S: Symptomatic; (*) One-sided, 97.5% CI, A: Asymptomatic.

**Table 2:** Univariable and multivariable logistic regression modeling of asymptomatic infections with SARS-CoV-2 (*n* = 328).

	Unadjusted		Adjusted (*n* = 179)	
Demographics	Odds Ratio	95% CI	Odds Ratio	95% CI
**Age (years)** _(S = 265) (A = 63)_				
Youth ^18–24^ (RC)	1.0			
Young Adults ^25–44^	3.1 **	1.3–7.3	2.2	0.8–5.8
Adults ^45–64^	0.7	0.3–2.1	0.7	0.2–2.3
Seniors ^65+^	4.0	1.0–16.8	6.9 *	1.3–35.8
**Sex** _(S = 265) (A = 63)_				
Male (RC)	1.0			
Female	0.8	0.8–2.3	1.2	0.6–2.2
**Ethnicity** _(S = 199) (A = 60)_				
Caucasian (RC)	1.0			
Asian	3.0 **	1.4–6.5	2.8 **	1.2–6.4
African	3.1	0.7–13.5	2.2	0.4–12.5
Middle Eastern	3.1	0.5–18.2	3.0	0.4–20.6
Other	4.7 **	2.2–10.1	4.5	2.0–10.2
**Occupation** _(S = 222) (A = 58)_				
Healthcare worker (RC)	1.0			
Essential service worker	4.3	0.9–20.2		
Residential age care worker	2.3	0.3–17.6		
Other working	2.3	0.5–11.2		
Other not working	6.5 *	1.1–38.0		
Other unknown	11.6 ***	2.6–52.7		
**Living situation** _(S = 254) (A = 63)_				
Alone (RC)	1.0			
With Family	0.7	0.2–2.5		
With Friends/Housemates	1.9	0.5–7.5		
**Comorbidity** _(S = 265) (A = 63)_				
None reported (RC)	1.0			
Yes	0.9	0.3–2.1		
**Smoker status** _(S = 99) (A = 11)_				
Never (RC)	1.0			
Current	0.5	0.1–3.9		
Former	1.0			
**Exposure type** _(S = 183) (A = 47)_				
Other (RC)	1.0			
Household	0.6	0.2–1.5		
Workplace	0.6	0.2–1.6		

Note. RC: reference category; **CI**: confidence interval; *** *p* < 0.001, ** *p* < 0.01, * *p* < 0.05; S: Symptomatic; A: Asymptomatic.

**Table 3:** Proportion of SARS-CoV-2 cases according to ethnicity, stratified by symptom profile (*n* = 259).

	Symptom Profile *n* (%)		
Ethnicity	Asymptomatic	Symptomatic	Total
Caucasian	21 (13.7%)	132 (86.3%)	153
Southeast Asian	7 (28.0%)	18 (72.0%)	25
Northeast Asian	7 (41.2%)	10 (58.8%)	17
Southern and Central Asian	2 (28.6%)	5 (71.4%)	7
African	3 (33.3%)	6 (66.7%)	9
Middle Eastern	2 (33.3%)	4 (66.7%)	6
Other minorities	18 (42.9%)	24 (57.1%)	42
Total	60	199	259

## Data Availability

The data presented in this study are available on request from the data custodian (D.C.). The data are not publicly available due to ethical reasons of the approving institute.

## References

[1] World Health Organisation (2018). Managing Epidemics: Key Facts about Major Deadly Disease.

[2] Cheng H.Y., Jian S.W., Liu D.P., Ng T.C., Huang W.T., Lin H.H. (2020). Taiwan COVID-19 Outbreak Investigation Team. Contact Tracing Assessment of COVID-19 Transmission Dynamics in Taiwan and Risk at Different Exposure Periods Before and After Symptom Onset. JAMA Intern. Med..

[3] World Health Organisation Coronavirus Disease. https://www.who.int/emergencies/diseases/novel-coronavirus-2019.

[4] Peter Vuillermin (Barwon Health, Geelong, VIC, Australia); Eugene Athan (Barwon Health, Geelong, VIC, Australia). The Barwon Health & Deakin University COVID-19 Research Task Force & Cohort Study—A data collection and biobank study of the region conducted through the early stage of the pandemic, 2020. (Unpublished work).

[5] Gao Z., Xu Y., Sun C., Wang X., Guo Y., Qiu S., Ma K. (2021). A systematic review of asymptomatic infections with COVID-19. J. Microbiol. Immunol..

[6] Kronbichler A., Kresse D., Yoon S., Lee K.H., Effenberger M., Shin J.I. (2020). Asymptomatic patients as a source of COVID-19 infections: A systematic review and meta-analysis. Int. J. Infect. Dis..

[7] Syangtan G., Bista S., Dawadi P. (2020). Asymptomatic SARS-COV-2 Carriers: A Systematic Review and Meta-Analysis. Front. Public Health.

[8] Roder C., Maggs C., McNamara B., O’Brien D., Wade A.J., Bennett C., Pascol J.A., Athan E. (2020). Area-level social and economic factors and the local incidence of SARS-CoV-2 infections in Victoria during. Med. J. Aust..

[9] Voysey M., Costa Clemens S.A., Madhi S.A., Weckx L.Y., Folegatti P.M., Aley P.K., Angus B., Baillie V.L., Barnabas S.L., Bhorat Q.E. (2021). Single-dose administration and the influence of the timing of the booster dose on immunogenicity and efficacy of ChAdOx1 nCoV-19 (AZD1222) vaccine: A pooled analysis of four randomised trials. Lancet.

[10] Augusto D.G., Murdolo L.D., Chatzileontiadou D.S.M., Sabatino J.J., Yusufali T., Peyser N.D., Butcher X., Kizer K., Guthrie K., Murray V.W. (2023). A common allele of HLA is associated with asymptomatic SARS-CoV-2 infection. Nature.

[11] Ravindra K., Malik V.S., Padhi B.K., Goel S., Gupta M. (2022). Asymptomatic infection and transmission of COVID-19 among clusters: Systematic review and meta-analysis. Public Health.

[12] StataCorp Stata Statistical Software Release 17. https://www.stata.com/.

[13] World Health Organisation Life Course. https://www.who.int/our-work/life-course.

[14] Ranganathan P., Pramesh C.S., Aggarwal R. (2017). Common pitfalls in statistical analysis: Logistic regression. Perspect. Clin. Res..

[15] Wang H., Peng J., Wang B., Lu X., Zheng J.Z., Wang K., Tu X.M., Feng C. (2017). Inconsistency Between Univariate and Multiple Logistic Regressions. Shanghai Arch. Psychiatry.

[16] Kang H. (2016). The prevention and handling of the missing data. Korean J. Anesthesiol..

[17] Byambasuren O., Cardona M., Bell K., Clark J., McLaws M.L., Glasziou P. (2020). Estimating the extent of asymptomatic COVID-19 and its potential for community transmission: Systematic review and meta-analysis. J. Assoc. Med. Microbiol. Infect. Dis. Can..

[18] Rentsch C.T., Kidwai-Khan F., Tate J.P., Park L.S., King J.T., Skanderson M., Hauser R.G., Schultze A., Jarvis C.I., Holodniy M. (2020). Patterns of COVID-19 testing and mortality by race and ethnicity among United States veterans: A nationwide cohort study. PLoS Med..

[19] Esteban I., Bergero G., Alves C., Bronstein M., Ziegler V., Wood C., Caballero M.T., Wappner D., Libster R., Perez Marc G. (2022). Asymptomatic COVID-19 in the elderly: Dementia and viral clearance as risk factors for disease progression. Gates Open Res..

[20] White E.M., Santostefano C.M., Feifer R.A., Kosar C.M., Blackman C., Gravenstein S., Mor V. (2020). Asymptomatic and Presymptomatic Severe Acute Respiratory Syndrome Coronavirus 2 Infection Rates in a Multistate Sample of Skilled Nursing Facilities. JAMA Intern. Med..

[21] Li L., Han Z.G., Qin P.Z., Liu W.H., Yang Z., Chen Z.Q., Li K., Xie C.J., Ma Y., Wang H. (2022). Transmission and containment of the SARS-CoV-2 Delta variant of concern in Guangzhou, China: A population-based study. PLoS Neglected Trop. Dis..

[22] Yanes-Lane M., Winters N., Fregonese F., Bastos M., Perlman-Arrow S., Campbell J.R., Menzies D. (2020). Proportion of asymptomatic infection among COVID-19 positive persons and their transmission potential: A systematic review and meta-analysis. PLoS ONE.

[23] Kumar N., Shahul Hameed S.K., Babu G.R., Venkataswamy M.M., Dinesh P., Kumar Bg P., John D.A., Desai A., Ravi V. (2021). Descriptive epidemiology of SARS-CoV-2 infection in Karnataka state, South India: Transmission dynamics of symptomatic vs. asymptomatic infections. EClinicalMedicine.

[24] Niedzwiedz C.L., O’Donnell C.A., Jani B.D., Demou E., Ho F.K., Celis-Morales C., Nicholl B.I., Mair F.S., Welsh P., Sattar N. (2020). Ethnic and socioeconomic differences in SARS-CoV-2 infection: Prospective cohort study using UK Biobank. BMC Med..

[25] Sze S., Pan D., Nevill C.R., Gray L.J., Martin C.A., Nazareth J., Minhas J.S., Divall P., Khunti K., Abrams K.R. (2020). Ethnicity and clinical outcomes in COVID-19: A systematic review and meta-analysis. EClinicalMedicine.

[26] Treibel T.A., Manisty C., Burton M., McKnight Á., Lambourne J., Augusto J.B., Couto-Parada X., Cutino-Moguel T., Noursadeghi M., Moon J.C. (2020). COVID-19: PCR screening of asymptomatic health-care workers at London hospital. Lancet.

[27] Olmos C., Campaña G., Monreal V., Pidal P., Sanchez N., Airola C., Sanhueza D., Tapia P., Muñoz A.M., Corvalan F. (2021). SARS-CoV-2 infection in asymptomatic healthcare workers at a clinic in Chile. PLoS ONE.

[28] Yoon S., Li H., Lee K.H., Hong S.H., Kim D., Im H., Rah W., Kim E., Cha S., Yang J. (2020). Clinical Characteristics of Asymptomatic and Symptomatic Pediatric Coronavirus Disease 2019 (COVID-19): A Systematic Review. Medicina.

[29] Griffith G.J., Morris T.T., Tudball M.J., Herbert A., Mancano G., Pike L., Sharp G.C., Sterne J., Palmer T.M., Smith G.D. (2020). Collider bias undermines our understanding of COVID-19 disease risk and severity. Nat. Commun..

[30] Le T.Q.M., Takemura T., Moi M.L., Nabeshima T., Nguyen L.K.H., Hoang V.M.P., Ung T.H.T., Le T.T., Nguyen V.S., Pham H.Q.A. (2020). Severe Acute Respiratory Syndrome Coronavirus 2 Shedding by Travelers, Vietnam. Emerg. Infect. Dis..

